# Non-surgical treatment of strabismus in children: a review of recent advances

**DOI:** 10.3389/fmed.2025.1582284

**Published:** 2025-06-27

**Authors:** Shulan Huang, Xuemei Zhong, Chanjuan Quan, Mingwen Zhang

**Affiliations:** ^1^Department of Ophthalmology, Xiaolan People's Hospital of Zhongshan (The Fifth People's Hospital of Zhongshan), Zhongshan, China; ^2^Department of Neurosurgery, Cranial Nerve Group, Xiaolan People's Hospital of Zhongshan (The Fifth People's Hospital of Zhongshan), Zhongshan, China

**Keywords:** strabismus, children, non-surgical treatment, corrective glasses, botulinum toxin

## Abstract

Pediatric strabismus is a progressive condition that, in the early stages, presents as either esotropia or intermittent or constant exotropia when both eyes focus on the same target. If not promptly addressed, the child’s ocular accommodative convergence and fusion abilities will gradually weaken, ultimately affecting visual function and causing various inconveniences in daily life and learning. For children with mild strabismus, those who are young, have poor cooperation, or are awaiting surgery for a long time, non-surgical treatment is a good option. It offers high safety, ease of use, and has certain effects in alleviating strabismus symptoms and improving visual function. However, there are multiple non-surgical treatment options, and currently, clinical practice lacks clear consensus on which approach is best. The choice and implementation of treatment methods still need to be determined based on the specific situation, such as the child’s age and type of strabismus. In recent years, with the deepening of ophthalmological research, non-surgical treatment methods have been enriched, including a combination of visual training, occlusion therapy, and corrective glasses, which have shown certain clinical effects. This article will review the non-surgical treatment options for pediatric strabismus, explore current research progress, and discuss future development directions.

## 1 Introduction

Strabismus is a common and prevalent eye disorder in children, with a global prevalence of approximately 3–5%. It not only affects the appearance of the child but may also lead to abnormal binocular visual function, dizziness, nausea, blurred vision, and other symptoms, impacting walking and balance (1). Herlihy et al. (2) reported that in the early stages of the disease, most children with strabismus can maintain proper eye alignment for a significant period. However, if left uncorrected, as the disease progresses, the child’s visual cortex perceptual system will gradually become disordered. Over time, retinal inhibition dark spots and retinal abnormalities will gradually form, eventually leading to a decline in binocular single vision in children. This can cause significant long-term inconvenience in the child’s daily life and learning, and in severe cases, it may even have a negative impact on the child’s psychological health. Therefore, early correction of strabismus is essential to maximize visual development during the critical period of visual maturation. Currently, surgical corrections such as muscle weakening, muscle strengthening, and horizontal muscle vertical displacement surgeries are the most effective methods for treating pediatric strabismus. These surgeries work by adjusting the length and insertion points of the extraocular muscles, altering the pulling forces on the eyeball to correct the strabismus. The goal is to prevent further deepening of retinal suppression scotomas and to improve the child’s binocular single vision function (3–5). However, due to factors such as the child’s young age, poor tolerance, and low cooperation, many parents of children with strabismus are often resistant to surgery and tend to opt for non-surgical treatment methods (6). In recent years, with the continuous advancement of medical technology, an increasing number of non-surgical treatments have been applied in the management of pediatric strabismus, yielding certain positive results. This review aims to summarize the latest research progress on non-surgical treatments for pediatric strabismus.

## 2 Pathogenesis of pediatric strabismus

At present, the pathogenesis of strabismus in children has not been fully elucidated. It is believed that it is related to various factors such as abnormal development of central nervous system, ametropia and unilateral visual input disorder. It has been reported in the literature that the normal movement of human extraocular muscles requires the accurate control of the central nervous system. Children are young, the development of the central nervous system is not yet mature, and the ability to control and regulate the extraocular muscles is weak, which may affect the normal movement of the eyeball, causing ptosis, intraocular rotation, downward rotation and upward rotation limitation, and eventually forming strabismus (7, 8). Secondly, some studies have also pointed out that when there is a significant difference in the refractive status of human eyes, the brain will inhibit the visual information of the eyes with higher refractive error, so as not to produce blurred visual interference. Long-term inhibition will lead to abnormal extraocular muscle function and eventually lead to strabismus. These children have a good response to refractive correction treatment (9, 10). In addition, it takes a long time for children ‘s binocular single vision function to gradually develop and improve after birth, and this process is relatively fragile. If it is affected by long-term insufficient light and non-standard eye use during this process, it may hinder the normal development of binocular single vision function and lead to the occurrence of strabismus (11). Non-surgical methods such as visual training, refractive correction, and neurotrophic drugs can safely and effectively promote the development of the central nervous system in children, guide the binocular work together, correct refractive errors, help the natural development of binocular monocular function, and promote the improvement of strabismus in children. This also provides a sufficient theoretical basis for the choice of non-surgical treatment options for strabismus in children (12).

## 3 Non-surgical treatment methods for pediatric strabismus

Although previous studies have confirmed that surgical correction of eye position is currently the most effective treatment for pediatric strabismus (13, 14), surgery carries certain risks, such as long waiting times and the potential for trauma. Moreover, for children, whose physical and mental development is still immature, undergoing surgery involves both psychological and physiological stress responses. These stress reactions can not only affect the success of the surgery itself but may also have negative impacts on the child’s behavior and psychological well-being. For young children with mild strabismus, poor cooperation during examination, or long waiting times for surgery, non-surgical treatments can be a good alternative option (15). Common non-surgical methods for pediatric strabismus include correction of refractive errors, using prisms, wearing bifocal lenses, occlusion therapy, orthoptic exercises, and botulinum toxin injections (Table 1). These treatments are simple to administer, well-tolerated by children, and generally accepted by parents. They have proven effective in improving binocular visual coordination, enhancing appearance and visual function, and reducing the degree of strabismus.

**Table 1:** Summary of key studies related to strabismus.

Author	Literature	Study type	Sample size	Intervention measures	Intervention results
Anilkumar et al.	(21)	Retrospective descriptive research	31 cases	Wearing a prism for intervention	87% of patients experienced disappearance of strabismus and diplopia, meeting the criteria for cure.
Summers et al.	(22)	Randomized controlled trial	57 cases, divided into prism group (*n* = 28) and non prism group (*n* = 29)	Prism group receives treatment with prism glasses, while non prism group receives treatment without prism glasses	After 8 weeks of intervention, the average baseline distance scores between the prism group and the non prism group were 3.3 and 3.6 points, respectively. The prism group showed improvement compared to before the intervention and was superior to the non prism group.
Tejedor et al.	(24)	Concept validation research	35 cases, divided into atropine group (*n* = 19) and atropine combined with bifocal group (*n* = 16)	The atropine group was treated only with atropine, while the atropine combined with bifocal group was treated with atropine and wearing bifocal lenses	After 6 months of intervention, the logMAR visual acuity of the atropine combined with bifocal group was (0.69 ± 0.15), which was lower than that of the atropine group (0.82 ± 0.20). The improvement in visual acuity was more significant in the atropine combined with bifocal group
Song et al.	(27)	Meta-Analysis	617 cases, observation group 313 cases, PTO group 304 cases	The observation group did not receive any intervention, while the PTO group received coverage therapy intervention (2–4 h/day)	Compared with the observation group, the PTO group showed a greater decrease in control of distant and near exotropia, and a more significant improvement in near stereoscopic visual acuity.
Tian et al.	(33)	Randomized controlled research	108 cases, divided into a training group (*n* = 78) and an untrained group (*n* = 30)	The training group received neuroplasticity training within 1 month after binocular strabismus correction surgery, while the untrained group only received binocular strabismus correction surgery	One month after surgery, the visual perception of the training group was better than that of the untrained group, and the visual quality was higher than that of the untrained group.
Zhang et al.	(34)	Randomized controlled research	200 cases, divided into a training group (*n* = 100) and a control group (*n* = 100)	The training group received virtual reality technology visual axis correction training within 1 week after surgery for common strabismus, while the control group did not attempt any training after surgery	After 6 months of postoperative follow-up, the training group had significantly higher orthorectified rates (degree of strabismus<8Δ) than the control group, and the number of individuals with simultaneous vision and remote stereoscopic vision was also significantly higher than the control group.
Niyaz et al.	(36)	Retrospective comparative research	86 cases	Treatment with injection of botulinum toxin type A	The success rate of treatment for esotropia was 31%, the success rate of treatment for partially regulated esotropia was 25%, and the success rate of treatment for residual esotropia was 61.5%.
Tugcu et al.	(37)	Retrospective comparative research	50 cases	Treatment with injection of botulinum toxin type A	The average deviation angle of strabismus after treatment decreased from (42.5 ± 13.2) PD before treatment to (12.8 ± 11.9) PD, and 60% of patients successfully achieved strabismus within 10 PD
Nguyen MTB	(38)	Multi center, retrospective research	76 cases, including 44 cases in the BTX group and 32 cases in the surgical group	The BTX group received injection of botulinum toxin type A for treatment, while the surgical group underwent strabismus surgery for treatment	At a follow-up of 36 months, the success rate of treatment in the BTX group was 72%, higher than the 56% in the surgical group, and the median deviation and median stereoscopic acuity were similar between the two groups.
Wang et al.	(39)	Retrospective comparative research	98 cases, including 28 cases in the BTX-A group, 25 cases in the BMR rc group, and 45 cases in the R&R group	The BTX-A group received bilateral injection of botulinum toxin type A into the rectus muscle, the BMR rc group received bilateral rectus muscle retraction surgery, and the R&R group received unilateral rectus muscle retraction combined with rectus muscle resection surgery	The success rate of movement in the BTX-A group during fixed distance and close distance was lower than that in the BMR rc group and R&R group. No children in the BTX-A group experienced overcorrection, while 4 and 20% of children in the R&R group and BMR rc group, respectively, developed continuous exotropia. There was no statistical significant difference in sensory results among the three groups of children
Marciano et al.	(40)	Retrospective single center research	There were 68 cases, which were divided into two subgroups: transconjunctival injection or open sky injection	The transconjunctival injection group was injected with botulinum toxin type A through the conjunctiva, while the open sky injection group was injected with botulinum toxin type A through the open sky	There was no significant difference in osteogenic rate between two injection methods for treating pediatric strabismus.

### 3.1 Correction of refractive errors

Excessive convergence response or imbalance of extraocular muscle strength caused by hyperopia is a major cause of strabismus in children (16). By accurately determining the refractive error of the child, corrective glasses with the appropriate prescription can effectively address refractive issues. This ensures that light focuses correctly on the retina, reduces the accommodative effort required for clear vision, and alleviates excessive convergence. At the same time, it improves the clarity and balance of visual input from both eyes. Clear visual stimuli help promote normal retinal and visual cortex development, restore normal binocular vision, and reduce the degree of strabismus (17, 18). Additionally, after correction of refractive errors for refractive errors, the child’s visual function improves, and the visual feedback mechanism helps make the movements of the extraocular muscles more coordinated and balanced. This allows both eyes to work together more effectively, further improving the child’s strabismus symptoms. Jones-Jordan et al. (19) analyzed the data of ametropic children wearing and not wearing frame glasses in four randomized controlled trials. The results showed that wearing frame glasses was beneficial to improve children’s stereoacuity. It can be seen that wearing frame glasses may improve the stereoacuity and visual function of children by correcting their ametropia. However, there are many types of strabismus, including accommodative strabismus and sensory strabismus. Whether different types of strabismus can benefit from wearing frame glasses is still lack of systematic research and analysis, and more research is needed in the future.

### 3.2 Prismatic correction

Since the mid-19th century, some Western countries have begun using prisms as an adjunctive treatment for strabismus. With the continuous development of technology, the manufacturing techniques of prisms have also been constantly improved. Currently, both domestically and internationally, adhesive prisms are primarily used as an adjunctive treatment for strabismus. These prisms consist of a set of Fresnel micro-structured prisms arranged in an orderly fashion. By altering the direction of light propagation, they help adjust the abductive fusion ability in strabismus patients, bringing the visual images on the retina closer together. This gradually increases the tension of the lateral rectus muscle, achieving a new balance between the tension of the lateral and medial rectus muscles. As a result, it alleviates visual disturbances and diplopia symptoms caused by strabismus, and improves the patient’s visual function. Prismatic correction can be used as a long-term management option, particularly in patients who are not suitable for surgical intervention or prefer non-invasive treatment. However, for patients with a large deviation angle, prism correction may be limited by the optical and cosmetic effects of high-power prisms. In such cases, prisms are usually used as a temporary or adjunctive measure prior to surgery (20). In addition, compared with the traditional glass prism, the pressed prism has the advantages of light structure and high correction accuracy, and is more suitable for younger children with small angle strabismus. Anilkumar et al. (21)conducted a retrospective case study, collecting data from 31 patients who received prism treatment for strabismus-related diplopia. The results showed that during a 3-year follow-up period, 81% of the patients experienced complete resolution of both strabismus and diplopia, meeting the criteria for cure. Summers et al. (22)randomly assigned 57 children aged 3–13 years with intermittent strabismus to a prism glasses group (*n* = 28) and a non-prism glasses group (*n* = 29). The average baseline distance control score for both groups was 3.5 before the intervention. After 8 weeks of intervention, the results showed that the average baseline distance control scores in the prism glasses group and non-prism glasses group were 3.3 and 3.6, respectively. The prism glasses group showed improvement compared to baseline and performed better than the non-prism glasses group. The studies mentioned above all suggest that adhesive prisms have a definite effect in improving the degree of strabismus and visual function in patients. However, these studies had relatively small sample sizes and were all conducted in single centers, which may affect the generalizability of the results. Future research should aim to increase the sample size and include multi-center studies to further confirm the effectiveness of adhesive prisms in the treatment of pediatric strabismus.

### 3.3 Wearing bifocal lens

The occurrence of strabismus in children (especially accommodative esotropia) is often related to abnormal ocular regulation function. When the ocular regulation function is abnormal, the eye may increase the convexity of the lens when looking at the object, causing the eye to over-collect and form strabismus. The bifocal lens refers to a special lens with different diopters in the upper part and the lower part of the lens. The upper part can maintain the diopter when looking at a distance, avoiding blurred vision caused by insufficient refractive adjustment. The convex lens in the lower part can reduce the adjustment demand when the eye is looking close, so as to meet the adjustment demand of the eye muscle by changing the focus position of the light, reduce the collective impulse of the eyeball, and alleviate esotropia (23). In addition, long-term wearing of bifocal lenses can also help the brain visual center to reintegrate binocular information by stabilizing retinal imaging, improve binocular coordinated motor function, promote the natural development of binocular single vision function, and ultimately improve the strabismus degree and visual function of children. A conceptual validation study divided 4-8-year-old children with strabismic amblyopia into atropine group (*n* = 19) and atropine combined with bifocal group (*n* = 16). The results showed that during the 6-month follow-up period, bifocal lenses combined with atropine treatment had better visual acuity improvement in children (atropine combined with bifocal group logMAR visual acuity 0.69 ± 0.15 VS atropine group 0.82 ± 0.20) (24). It can be seen that wearing bifocal lenses can promote the improvement of visual acuity in children with strabismus. However, most of the existing studies on the treatment of children’s strabismus with bifocal lenses are small sample studies or combined with other interventions. There is a lack of large-scale randomized controlled studies directly on the treatment of children’s strabismus with bifocal lenses. The effect of its application alone still needs to be verified by more high-quality randomized controlled studies.

### 3.4 Occlusive patching

Studies have pointed out that in order to avoid diplopia, the brain will actively inhibit the visual input of strabismus eyes. In the long run, it will lead to poor vision and abnormal fixation function, forming a vicious circle and increasing the degree of strabismus (25). The occlusion therapy can force the strabismus eye to receive visual stimulation by blocking the dominant eye (healthy eye), thereby activating the remodeling of the visual cortex neurons, improving the visual signal processing ability of the strabismus eye, and reducing the competitive inhibition of the healthy eye on the strabismus eye, thereby improving the binocular visual axis offset, promoting the development of binocular fusion function, and ultimately improving the visual function of the body (26). A meta-analysis included 4 randomized controlled studies, a total of 617 cases of intermittent exotropia in children with data, analysis of the application of occlusion therapy in the treatment of children with simple observation and follow-up of children with strabismus control effect, the results showed that compared with the simple observation and follow-up of children with, receiving occlusion therapy in children with distant and near exotropia angle was significantly decreased, the distance deviation was significantly reduced, near stereoacuity was significantly improved (27). It can be seen that occlusion therapy also has a good effect in improving the strabismus angle and near stereoacuity of children with strabismus. However, the duration and frequency of occlusion therapy will also affect the intervention effect of pediatric strabismus. At present, there is still a lack of large-sample randomized controlled studies to conduct in-depth analysis. In the future, more studies are needed to further clarify the optimal occlusion duration and frequency of pediatric strabismus.

### 3.5 Orthoptic exercises

Under normal conditions, the retinas of both eyes have a precise correspondence, allowing the images seen by both eyes to fuse accurately in the brain, forming a single, clear, and stereoscopic visual perception. However, when strabismus occurs, the directions of the eyes’ gaze are misaligned, leading to a disruption in the retinal correspondence between the two eyes. As a result, the images seen by both eyes cannot be fused into a single, clear image in the brain, ultimately causing symptoms such as diplopia, visual fatigue, dizziness, and nausea (28, 29). orthoptic exercises uses specific techniques such as visual stimulation therapy and binocular coordination training to help strabismus patients focus both eyes on a particular image or target simultaneously. During the training process, parameters such as the image’s position, angle, and size are gradually adjusted. This approach aims to enhance binocular fusion ability and guide the retinas of both eyes to re-establish normal correspondence. Over time, the brain adapts and learns to properly process the visual information from both eyes, ultimately improving the degree of strabismus and enhancing the patient’s visual function (30, 31). Additionally, orthoptic exercises can also include a series of eye movement exercises, such as ocular tracking and saccadic training, to strengthen the power and flexibility of the extraocular muscles. These exercises enhance the coordination between the extraocular muscles of both eyes, improving the accuracy and stability of eye movements, which helps reduce the degree of strabismus (32). Tian et al. (33)divided 108 strabismus patients into a training group (78 patients) and a non-training group (30 patients) based on whether they received visual neuroplasticity training post-surgery. The results showed that the training group had better visual perception and visual quality compared to the non-training group. In a randomized controlled trial conducted by Zhang et al. (34), 200 strabismus patients who had undergone surgery were divided into a training group and a control group, with 100 patients in each group. The training group received orthoptic exercises using virtual reality technology, while the control group did not undergo any training. The results showed that after 6 months of intervention, the training group had a significantly higher rate of orthophoria (with near or distant strabismus < 8^Δ^) compared to the control group. Additionally, the number of patients in the training group who achieved both simultaneous vision and distance stereopsis was also significantly higher than in the control group. It can be seen that orthoptic exercises can help improve the degree of strabismus and visual function in children with strabismus. However, there are various methods of orthoptic exercises for pediatric strabismus, and there is currently no unified clinical standard to regulate these approaches. Future research should focus on further standardizing the indications and contraindications for different types of orthoptic exercises, ensuring the safety of the children while maximizing the effectiveness of the intervention.

### 3.6 Botulinum toxin type a treatment

Pharmacological treatment is also an important non-surgical method for treating pediatric strabismus. It primarily works by controlling eye movements, adjusting the strength of the extraocular muscles, or improving ocular nerve function, thereby correcting strabismus or alleviating its symptoms. Among these, Botulinum Toxin Type A is the most commonly used. Research has shown that Botulinum Toxin Type A is a bacterial exotoxin secreted by *Clostridium botulinum* during its growth process. It acts on the terminal ends of cholinergic motor neurons, interfering with the release of acetylcholine, which weakens the local muscle force and inhibits muscle contraction. When used in the treatment of strabismus, it can adjust the strength balance of the extraocular muscles, restore the normal position of the eyeball, and ultimately relieve the symptoms of strabismus (35). Niyaz et al. (36)included 86 strabismus patients in their study, all of whom received botulinum toxin treatment. The results showed a significant improvement in the degree of esotropia in these patients. Specifically, the cure rate for infantile esotropia was 31%, the cure rate for partially accommodative esotropia was 25%, and the cure rate for residual esotropia was 61.50%. In a study conducted by Tugcu et al. (37), 50 strabismus patients were selected as study subjects, all of whom received Botulinum Toxin Type A treatment. The results showed that the average strabismus deviation angle decreased from (42.5 ± 13.2) PD before treatment to (12.8 ± 11.9) PD after treatment. Additionally, 60% of the patients successfully achieved a strabismus deviation of less than 10 PD. Therefore, it is clear that Botulinum Toxin Type A plays an active role in the treatment of strabismus. In a multi-center, retrospective, non-randomized, comparative study conducted by Nguyen et al. (38), 76 children with acute acquired concomitant esotropia were selected as the research objects. According to different treatment methods, they were divided into botulinum toxin treatment group (BTX group, 44 cases) and strabismus surgery group (surgery group, 32 cases). The clinical efficacy of the two groups at 36 months after treatment was analyzed. The results showed that the success rate of treatment in the BTX group was 72%, which was higher than that in the surgery group (56%). The median deviation of the two groups was similar to the median stereoacuity. In a retrospective comparative clinical study conducted by Wang et al. (39), 98 children with partially accommodative esotropia were divided into BTX-A group (28 cases, bilateral medial rectus injection of botulinum toxin type A), BMR-rc group (25 cases, bilateral medial rectus recession) and R&R group (45 cases, unilateral medial rectus recession combined with lateral rectus resection) according to different treatment methods. The results showed that the success rate of movement in BTX-A group was lower than that in BMR-rc group and R&R group at long-distance and short-distance fixation, but no children in BTX-A group had excessive correction. There were 4 and 20% children with continuous exotropia in the R&R group and the BMR-rc group, respectively, and there was no statistical significant difference in sensory results among the three groups. In addition, there are many ways of administration of botulinum toxin type A. A retrospective single-center study analyzed the relevant data of children with strabismus who received injection of botulinum toxin type A by conjunctival injection and open sky injection, respectively. The results showed that there was no significant difference in the success rate of treatment of strabismus in children by conjunctival injection and open sky injection (40). It can be seen that botulinum toxin type A also plays an active role in the treatment of strabismus. It can obtain similar therapeutic effects as surgery, and no matter what kind of medication, it can obtain better therapeutic effects. However, some studies have found that excessive use of botulinum toxin type A may lead to adverse reactions such as ptosis, diplopia, and limited eye movement in children with strabismus, which will limit the effect of drug treatment to a certain extent and reduce the clinical benefits of children (41). Therefore, in the future, it is necessary to further standardize the optimal dosage of botulinum toxin type A in the treatment of pediatric strabismus through a large sample size prospective randomized controlled study, so as to increase the clinical benefits of children as much as possible while ensuring the safety of medication.

## 4 Conclusion

Pediatric strabismus is a common eye disorder that can cause abnormalities in both the appearance and visual function of the affected child, which negatively impacts their physical and psychological development as well as their ability to live and learn normally. Non-surgical treatments such as wearing corrective glasses, using adhesive prisms, wearing bifocal lenses, covering therapy, orthoptic exercises, and Botulinum Toxin Type A therapy have advantages over surgical treatment, including safety, minimal trauma, and higher acceptance from both children and parents. These methods can effectively and safely reduce the degree of strabismus and improve visual function through various mechanisms, such as correcting refractive errors, improving the strength and coordination of extraocular muscles, promoting local blood circulation, and stimulating the nerves. However, the effectiveness of the aforementioned non-surgical treatments varies depending on the duration of the disease in the patient, and the safety and treatment outcomes also differ with varying treatment frequencies and durations. In the future, it will be essential to further standardize and optimize non-surgical treatment plans for strabismus in order to maximize clinical efficacy while ensuring safety. This will help increase the clinical benefits for children and promote the relief or even complete resolution of strabismus symptoms.
